# ProLIF: a library to encode molecular interactions as fingerprints

**DOI:** 10.1186/s13321-021-00548-6

**Published:** 2021-09-25

**Authors:** Cédric Bouysset, Sébastien Fiorucci

**Affiliations:** Institut de Chimie de Nice UMR7272, Université Côte d’Azur, CNRS, Nice, France

**Keywords:** Interaction fingerprint, Structural biology, Molecular dynamics, Docking, Virtual screening, Python

## Abstract

Interaction fingerprints are vector representations that summarize the three-dimensional nature of interactions in molecular complexes, typically formed between a protein and a ligand. This kind of encoding has found many applications in drug-discovery projects, from structure-based virtual-screening to machine-learning. Here, we present ProLIF, a Python library designed to generate interaction fingerprints for molecular complexes extracted from molecular dynamics trajectories, experimental structures, and docking simulations. It can handle complexes formed of any combination of ligand, protein, DNA, or RNA molecules. The available interaction types can be fully reparametrized or extended by user-defined ones. Several tutorials that cover typical use-case scenarios are available, and the documentation is accompanied with code snippets showcasing the integration with other data-analysis libraries for a more seamless user-experience. The library can be freely installed from our GitHub repository (https://github.com/chemosim-lab/ProLIF).

**Supplementary Information:**

The online version contains supplementary material available at 10.1186/s13321-021-00548-6.

## Introduction

Interactions between and within molecular structures are the driving force behind biological processes, from protein folding to molecular recognition. The decomposition of interactions by residues in biomolecular complexes can provide insights into structure–function relationships, and characterizing the nature of each of these interactions can guide medicinal chemists in structure-based drug discovery projects [1]. Approaches to encode the interactions observed in 3D structural data in the form of a binary fingerprint have been developed in the past [2–6] and applied successfully to a variety of projects. For example, de Graaf et al. [7] used the Tanimoto similarity between the interaction fingerprint (IFP) of a crystallographic reference and the IFP of docking poses to rescore virtual screening results on a G protein-coupled receptor (GPCR). Rodríguez-Pérez et al. [8] showed that IFPs can achieve superior predictive performance than ligand fingerprints (ECFP4) for the classification of kinase inhibitor binding modes with machine-learning models. Finally, Mpamhanga et al. [9] showed that one can use the IFP for clustering, and then shortlist a reasonable number of binding modes prior to visual inspection. More recently, the approach was also implemented for molecular dynamics (MD) simulations to study ligand unbinding [10]. While the typical IFP usually encodes pre-established interactions (hydrogen bond, π-stacking…etc.) on a per-residue basis, other implementations exist. Sato et al. [11] developed a pharmacophore-based IFP which relies on the pharmacophoric features of the ligand atoms in contact with the protein and the distance between each of these pharmacophores to generate a bitvector. Da et al. [12] developed an IFP that relies on the atomic environment of both the protein and ligand interacting atoms to set the positions of a bit in the fingerprint, rather than relying on protein residues and predefined interactions, which has the advantage of implicitly encoding every possible type of interaction. This protocol was later reimplemented in Python by Wójcikowski et al. [13], but other more classical Python-based IFP implementations exist [14–19]. In this paper, we introduce a new Python library, ProLIF, that overcomes several limitations encountered by these programs, namely working exclusively with the output of specific docking programs, not being compatible with the analysis of MD trajectories, being restricted to a specific kind of complex (usually protein–ligand complexes), depending on residue or atom type naming conventions, or not being extensible or configurable regarding interactions (Table 1).

**Table 1:** Comparative table of features available in non-commercial IFP software

Software	Complexes	Input format	MD	Configurable	Extensible	Web	CLI	Ref.
ProLIF	All	MDAnalysis and RDKit^c^	✓	✓	✓			
IChem	LP	MOL2		✓			✓	[4]
PLIP	All^a^	PDB		✓		✓	✓	[15, 18]
Arpeggio	All^a^	PDB		✓		✓	✓	[16]
PyPLIF HIPPOS	LP^a^	PDBQT, MOL2		✓			✓	[17]
getContacts	All^a,b^	VMD^c^		✓			✓	[19]
MD-IFP	LP^a^	MDAnalysis^c^	✓					[10]
ODDT	LP	OpenBabel and RDKit^c^		✓				[20]

*MD* native support of MD trajectories; *Web* available through a webserver; *CLI* command-line interface; *LP* Ligand–Protein; *All* all combinations between ligand, protein, DNA, and RNA molecules

^a^Relies on residue and atom naming convention to assign charges and/or aromaticity

^b^Only supports hydrogen-bond, hydrophobic and van der Waals contacts for LP complexes

^c^Compatible with the input formats supported by the aforementioned libraries

## Implementation

ProLIF can deal with RDKit [21] molecules or MDAnalysis [22] Universe objects as input, which allows supporting most 3D molecular formats, from docking to MD simulations. While most MD topology files do not keep explicit information about bond orders and formal charges, MDAnalysis is able to infer this information if all hydrogen atoms are explicit in the structure while converting the structure to an RDKit molecule. The RDKit parent molecule is then automatically fragmented in child residue molecules based on residues name, number, and chain to make it easier to work on a per-residue basis when encoding the interactions.

When calculating an interaction fingerprint, each interaction is typically defined as two groups of atoms that satisfy geometrical constraints based on distances and/or angles (Table 2). Here the selection of atoms is made using SMARTS queries (Table 3), which is more precise than relying on elements or atomic weights and is also more universal than relying on force-field-specific atom types.

**Table 2:** Interactions currently available in ProLIF

Interaction	Ligand^a^	Protein^a^	Distance (Å)	Angle (deg)
Anionic	Anion	Cation	≤ 4.5	
Cationic	Cation	Anion		
CationPi	Cation	Aromatic	(+)-ctd ≤ 4.5	$\langle \overrightarrow{n}, \overrightarrow{ctd\cdots (+)}\rangle \in \left[0, 30\right]$
PiCation	Aromatic	Cation		
PiStacking	Aromatic	Aromatic	ctd–ctd ≤ 6.0	$\langle \overrightarrow{n}, \overrightarrow{n}\rangle \in \left[0, 90\right]$
			min ≤ 3.8	
EdgeToFace	Aromatic	Aromatic	ctd–ctd ≤ 6.0	$\langle \overrightarrow{n}, \overrightarrow{n}\rangle \in \left[50, 90\right]$
			Min ≤ 3.8	
FaceToFace	Aromatic	Aromatic	ctd–ctd ≤ 4.5	$\langle \overrightarrow{n}, \overrightarrow{n}\rangle \in \left[0, 40\right]$
			min ≤ 3.8	
HBAcceptor	HBAcceptor	HBDonor	D–A ≤ 3.5	$\langle \overrightarrow{HD}, \overrightarrow{HA}\rangle \in \left[130, 180\right]$
HBDonor	HBDonor	HBAcceptor		
XBAcceptor	XBAcceptor	XBDonor	X–A ≤ 3.5	$\langle \overrightarrow{XD}, \overrightarrow{XA}\rangle \in \left[130, 180\right]$
XBDonor	XBDonor	XBAcceptor		$\langle \overrightarrow{AX}, \overrightarrow{AR}\rangle \in \left[80, 140\right]$
MetalAcceptor	Ligand	Metal	≤ 2.8	
MetalDonor	Metal	Ligand		
Hydrophobic	Hydrophobic	Hydrophobic	≤ 4.5	

*(−)* anion; *(*+*)* cation; *ctd* centroid of the aromatic ring; *min* minimum value in the distance matrix between both aromatic rings; *n* normal to the aromatic ring plane; *D* hydrogen/halogen bond donor; *A* hydrogen/halogen bond acceptor; *H* hydrogen atom; *X* halogen atom; *R* atom linked to a halogen bond acceptor

^a^Although “ligand” and “protein” are used here, all the listed interactions can be applied to any molecular complex (protein–protein, DNA–protein…etc.)

**Table 3:** SMARTS patterns used in the definition of interactions

Name	SMARTS pattern(s)
Anion	[−{1−}]
Cation	[+{1−}]
Aromatic	a1:a:a:a:a:a:1
	a1:a:a:a:a:1
HBAcceptor	[N,O,F,−{1−};! + {1−}]
HBDonor	[#7,#8,#16][H]
XBAcceptor	[#7,#8,P,S,Se,Te,a;! + {1−}][*]
XBDonor	[#6,#7,Si,F,Cl,Br,I]-[Cl,Br,I,At]
Metal	[Ca,Cd,Co,Cu,Fe,Mg,Mn,Ni,Zn]
Ligand	[O,N,−{1−};! + {1−}]
Hydrophobic	[#6,#16,F,Cl,Br,I,At; + 0]

The library is designed so that users can easily modify existing interactions, as there is usually no consensus on the empirical thresholds (distance, angles) that should be used. For example, the hydrogen bond DH…A can be defined as a distance between H and A lower or equal to 3.0 Å [9] or as a distance between D and A lower or equal to 3.5 [4, 14, 20] or 4.1 Å [15, 23], and the angles constraints can also vary. ProLIF is also designed to let users define custom interactions.

Each interaction is written as a Python class that implements a “detect” method which takes two RDKit molecules as input, typically a ligand and a protein residue, and outputs a Boolean (True if the interaction is present, else False) as well as the indices of atoms responsible for the interaction. All interaction classes are then gathered inside a “Fingerprint” class that can generate a bitvector from two RDKit molecules, and optionally return the atom indices. By default, the Fingerprint class is configured to generate a bitvector with the following interactions: hydrophobic, π-stacking, π-cation and cation-π, anionic and cationic, and H-bond donor and acceptor, although more specific interactions are available (see Table 2). This Fingerprint class is designed with two scenarios in mind, post-processing MD trajectories or docking results, thus it provides user-friendly functions to generate the complete array of interactions for each pair of interacting residues.

Finally, the interaction is stored inside the Fingerprint class as a mapping between a pair of “ligand” and “protein” residues, and the corresponding interaction bitvector. For easier post-processing, the interaction fingerprint can then be converted to a pandas DataFrame object [24], which facilitates the search for specific interactions and the aggregation of results.

## Results and discussion

By relying on the interoperability with popular open-source libraries (MDAnalysis and RDKit), it can support a wide range of molecular formats typically found in docking experiments and MD simulations. Because it directly relies on SMARTS patterns to define the chemical moieties that partake in interactions, it is also compatible with any kind of molecular complex, including complexes made of ligands, proteins, DNA or RNA molecules. Interoperability also allows for data analysis to be substantially easier: as mentioned in the Implementation section the IFP can be directly exported to a pandas DataFrame (one of Python’s most popular data analysis library), and the documentation contains tutorials on how to visualize the interactions as graphs or how to display them on the 3D structure of the complex.

### Analysis of an MD trajectory of a GPCR in complex with a ligand

The code to run ProLIF on an MD trajectory can be as simple as follow:
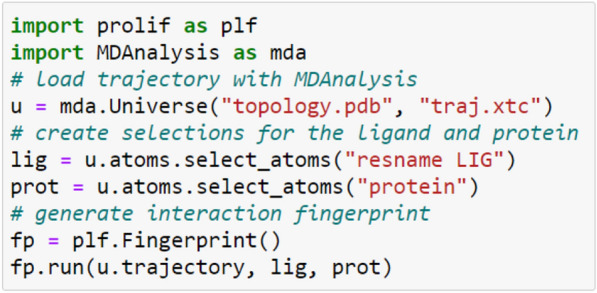


Here, we showcase an analysis based on the fingerprint obtained from a 500 ns MD simulation of the 5-HT1B receptor (class A aminergic GPCR) in complex with ergotamine retrieved from the GPCRmd webserver (id 90) [25]. In class A GPCRs, each position is annotated in superscript notation according to the Ballesteros-Weinstein numbering scheme [26], a generic residue numbering denoting both the helix and position relative to the most conserved residue labelled as number 50.

Exporting the fingerprint to a DataFrame allows to easily address common questions like which residues are involved in a specific type of interaction, which interactions does a specific residue do, which are the most frequent types of interactions, or which are the residues most frequently interacting with the ligand. In this MD trajectory, there is constantly at least one hydrophobic, H-bond donor and cationic interaction, while H-bond acceptor and π-stacking interactions occur respectively in 92% and 85% of the analyzed frames (see analysis notebook in supplementary data). F331^6.52^ is responsible for half of the π-stacking interactions occurring during the simulation, and the ten residues that interact with the ligand the most frequently are (in descending order): D129^3.32^, I130^3.33^, F330^6.51^, V201^ECL2.52^, F331^6.52^, S212^5.42^, W327^6.48^, V200^ECL2.51^, C133^3.36^ and F351^7.35^ which are all in contact with the ligand in at least 97% of frames. This is in agreement with the known interactions available from experimental structures as listed on the GPCRdb webpage [27] for the human 5-HT1B receptor, except for S212^5.42^ which isn’t reported to make H-bond interactions with ligands. The difference is likely due to the fact that this analysis is based on an MD trajectory while GPCRdb gathers interactions from experimental structures. However, GPCRdb also lists mutational data for S212^5.42^, and mutating this position to an alanine does not affect the binding affinity to ergotamine [28] which could coincide with the MD simulation since the ligand makes a hydrogen bond with the backbone and not the sidechain. Mutating S212^5.43^ to a bulkier residue could potentially affect this interaction and decrease the binding affinity.

**Fig. 1:**
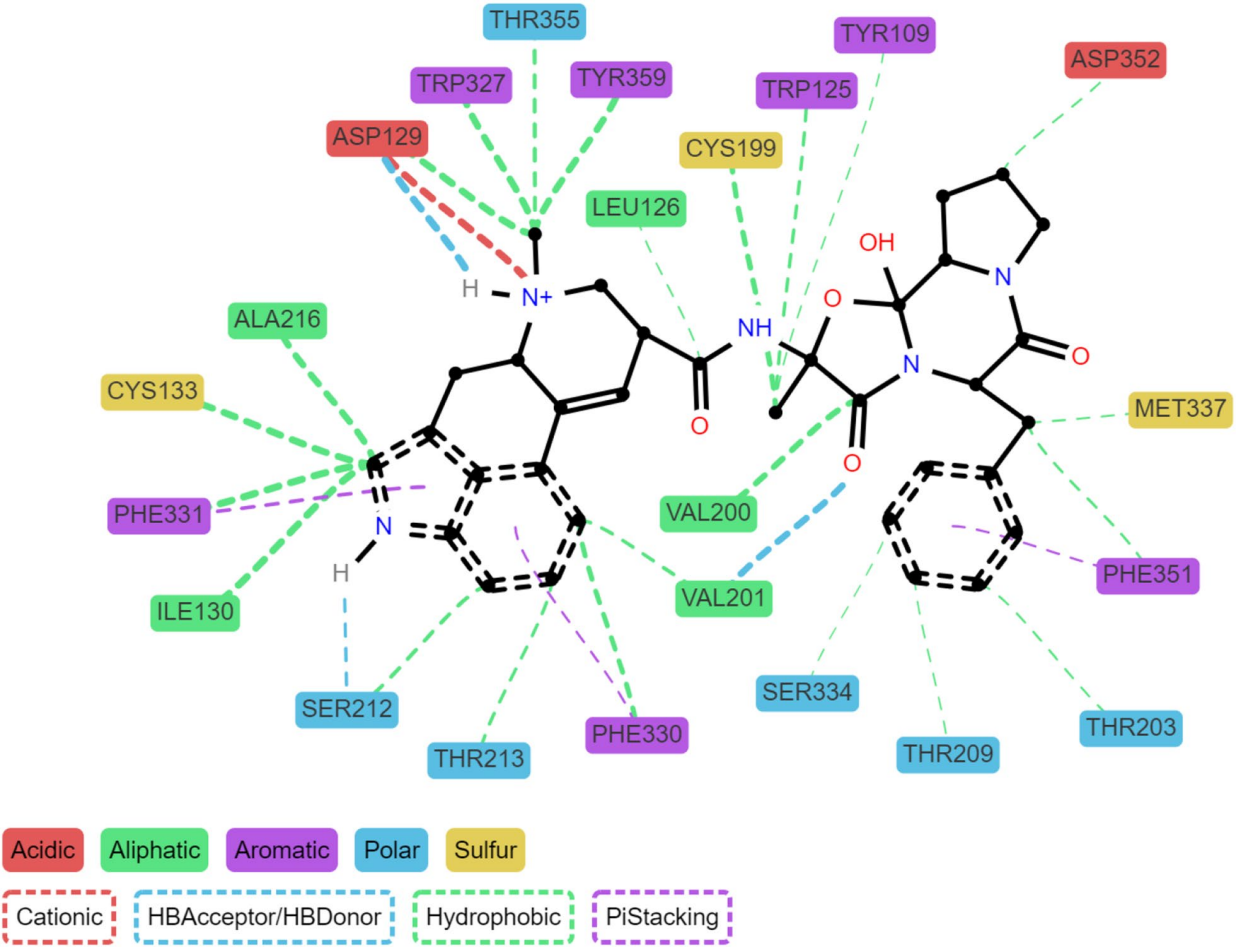
Ligand interaction network for the ergotamine agonist bound to the 5-HT1B receptor. Each interaction is shown as a dashed line between the residue and the ligand, and the width of the line is linked to the frequency of the interaction in the simulation. Only interactions occurring in at least 30% of frames are shown here

Because ProLIF keeps track of the atom indices responsible for interactions, it is possible to display detailed 2D or 3D interaction plots. Examples of scripts to generate such plots are given in the documentation. An exception is made for the ligand interaction network diagram which has been directly included in the source code of ProLIF under the LigNetwork class. This LigNetwork diagram (Fig. 1) is interactive and allows repositioning the residues but also hiding specific residue types or interactions by clicking the legend. It can show the interaction diagram at a precise frame or aggregate the results and only display interactions that appear frequently, controlled by a frequency threshold. In the latter case, to keep the plot readable for each ligand–protein-interaction group only the most frequent ligand atom is shown, as it might differ between frames.

**Fig. 2:**
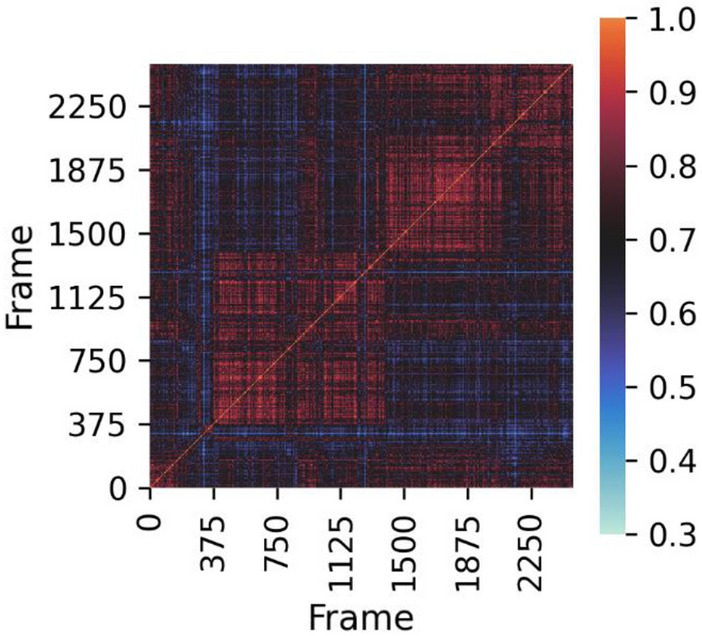
Tanimoto similarity matrix of ligand–protein interactions between each frame of the MD trajectory

The fingerprint can also be converted to an RDKit bitvector to make use of the similarity/distance metric functions implemented. This allows to investigate the presence of different binding modes in the simulation. In Fig. 2, we show the Tanimoto similarity matrix between each interaction fingerprint during the MD simulation. Two clusters are visible (from frame 400 to 1400, and from frame 1400 to 2100) which reveals changes in the interactions between ergotamine and 5-HT1B. Indeed, in the second cluster the phenyl ring of ergotamine gets closer to the indole moiety, which disrupts hydrophobic contacts with W125^3.28^, H-bonding with S212^5.42^ and π-stacking with F351^7.35^ to create new hydrophobic interactions with T203^ECL2^, T209^5.39^, S334^6.55^ and D352^7.36^.

### Analyzing protein–protein interactions (PPI)

The analysis of intra- and inter-molecular interactions can also be applied to investigate protein dynamics and function with ProLIF. Because ProLIF requires explicit hydrogen atoms, we preprocess PDB files of X-ray structures in the current section with the PDB2PQR [29] webserver as follows: AMBER force-field and naming scheme, protonation states assignments with PROPKA at pH 7.0, H-bond network optimization and removal of water molecules.

**Fig. 3:**
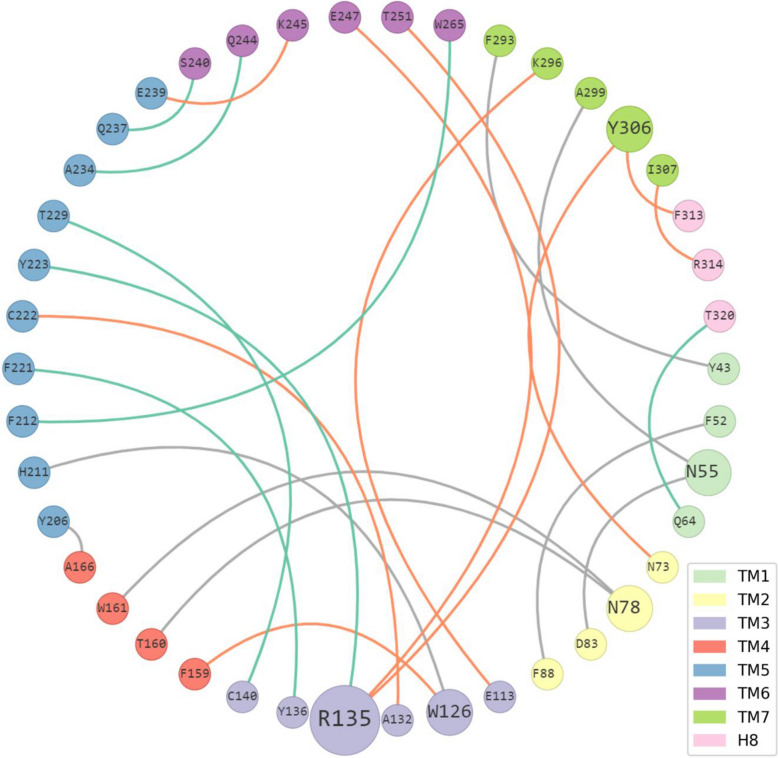
Residue interaction network for the bovine rhodopsin. Residues are colored by transmembrane domain (TM). Interactions that only appear in the active (PDB 6FK6) or inactive (PDB 1U19) state of the receptor are respectively shown in green or orange, and the ones that appear in both are in grey. Each residue node is scaled based on its number of interactions. For clarity, interactions that occur within the same TM (as labelled by GPCRdb) and interactions between residues that are less than 3 residues apart are not shown, as well as hydrophobic interactions (as defined in the implementation) and residues that did not participate in any interaction

In this first example, we focus on the activation mechanism of a class A GPCR and show how ProLIF can help pinpoint intramolecular structural modifications upon receptor activation. GPCRs are membrane-embedded receptors arranged in seven helical transmembrane domains (labelled TM1 to TM7) followed by a shorter helix (H8) that lies at the interface between the membrane and the cytosol. This family shares conserved key motifs in each TM domain, and some of the motifs are part of molecular switches that mediate ligand binding or receptor activation. Among them, the DRY motif in TM3 and the NPxxY motif in TM7 have been reported to be part of the allosteric mechanism [30]. Briefly, upon ligand binding, the signal propagates from the binding pocket to the ionic lock (comprised of the DRY motif) through a network of hydrophobic residues. The ionic lock maintaining the receptor in its inactive form is disrupted, leading to an increase of the inter-helix distances (notably TM3-TM6). At the same time, the hydrophobic barrier cannot prevent anymore the flooding of the intracellular part of the receptor thereby creating an intracellular crevice required for G protein coupling. R^3.50^ of the DRY motif is known to stabilize the inactive form of the rhodopsin receptor through a salt-bridge with D^6.30^ known as the “ionic-lock” [30]. This position can also interact with Y^5.58^ through an H-bond, and is reported to be critical for the formation of the active state in the β2 adrenergic receptor [31]. For the NPxxY motif, the mutation of Y^7.53^ disrupts interactions with N^2.40^ in the β2 adrenergic receptor [32], and Y^7.53^ is also reported to have an aromatic interaction with F^8.50^ which stabilizes the inactive conformation of the rhodopsin receptor [33]. As an example, the residue interaction network of the bovine rhodopsin in both active (PDB 6FK6) and inactive (PDB 1U19) states is studied to reveal the structural changes involving these two motifs. As seen in Fig. 3, the ionic lock between R135^3.50^ and E247^6.30^ is only visible in the inactive form of the receptor, while the interaction between R135^3.50^ and Y223^5.58^ was only detected in the active form. Y306^7.53^, which is part of the NPxxY motif in TM7, takes part in both key interactions that stabilize the inactive form of the receptor previously described: an H-bond interaction with N73^2.40^ and a π-stacking interaction with F313^8.50^. Finally, in rhodopsin, the salt-bridge between K296^7.43^ and E113^3.28^ is known to be crucial in the activation cycle of the receptor and is only disrupted when K296^7.43^ transiently bounds to retinal [34], which is in agreement with the interactions reported here.

**Fig. 4:**
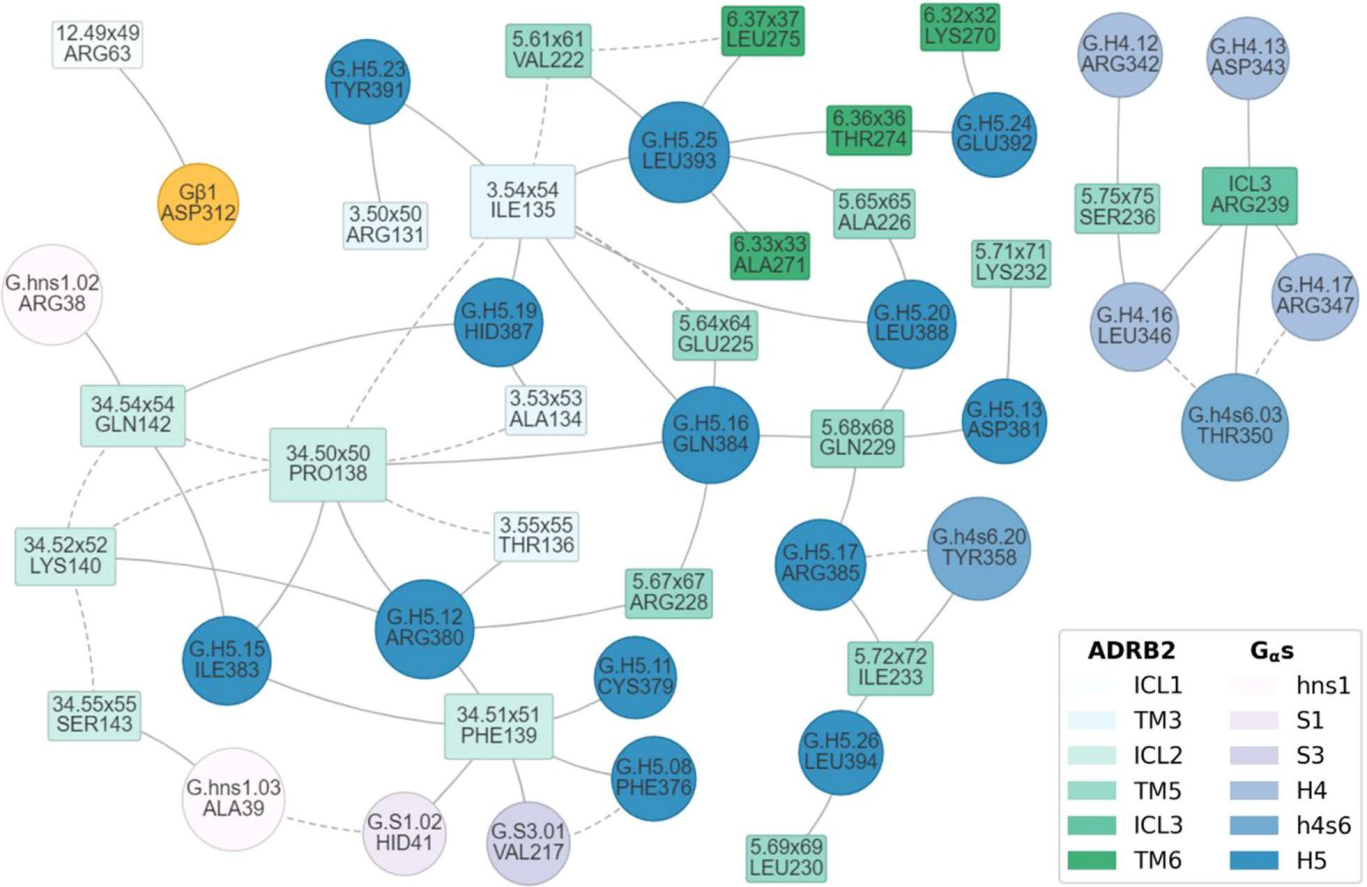
Interaction network between the β2 adrenoceptor (ADRB2) and G protein complex (Gαs and Gβ1). ADRB2 residues are shown as rectangles in shades of green, and G protein residues are shown as ellipses in shades of blue for Gαs and in yellow for Gβ1. For ADRB2, ICL denotes the intracellular loops while TM corresponds to the transmembrane domains. For Gαs, the common Gα numbering (CGN) system is used [37]. Each node is scaled by its number of interactions. Inter and intra protein interactions are respectively shown as plain and dashed lines. Residues that do not participate in GPCR-G protein interactions are not shown, and interactions between covalently bonded residues or residues of the same helix (as labelled by GPCRdb) are hidden

The final step in GPCR signal transduction being an intermolecular process between the GPCR and a G-protein, ProLIF can also be used in this case to highlight positions that dictate the coupling specificity in a series of GPCR-G-protein complexes. Here, we reproduce the analysis of interactions between the β2 adrenoceptor and the Gαs/Gβ1 complex by Flock et al. [35] where the authors used a “van der Waals contact” interaction based on Venkatakrishnan et al. [36] which considers two residues as interacting if any interatomic distance is below or equal to their van der Waals interaction distance (the sum of their van der Waals radii plus a tolerance factor of 0.6 Å). We reimplemented this in ProLIF (see analysis notebook in supplementary data) and applied it to the same structure (PDB 3SN6) to obtain the PPI network shown in Fig. 4.

The interaction network remains mostly the same as with Figure S6 of the original study [35] and highlights the importance of positions I135^3.54^, P138^34.50^, F139^34.51^, Q229^5.68^, R239^ICL3^ and T274^6.36^ for GPCR-G protein coupling. Using the default ProLIF implementation would help clarifying the types of interactions involved (H-bond, ionic…etc.) for a better understanding of coupling specificity when several GPCR-G protein complexes are investigated.

## Conclusions

ProLIF is a new Python library that overcomes limitations encountered by other freely available IFP programs. One of the main differences is the support of MD trajectories, while still being compatible with other molecular structure files like docking and experimental structures. By design, it is also not restricted to a particular kind of molecular complex but supports any combination of ligand, protein, DNA, or RNA molecules, thanks to its absence of dependency to force-field specificities such as atom types or residue naming convention. It also has a user-friendly API, comes with several tutorials, and allows creating custom interactions or reconfiguring existing ones. Finally, it focuses on the integration with typical data-analysis packages and visualization tools for a seamless user experience within the Python ecosystem. Possible improvements include the addition of more interactions types, but also more types of fingerprints such as the pharmacophoric [11] or circular [12, 13] fingerprints. Adding a command-line interface would also extend the userbase to researchers inexperienced in Python. Another point of interest could be the extension to other popular visualization libraries for a more streamlined data analysis experience for users.

## Availability and requirements

Project name: ProLIF.

Project home page: https://github.com/chemosim-lab/ProLIF

Operating system(s): platform independent.

Programming language: Python.

Other requirements: Python 3.6 or higher, and several open-source Python packages listed in the project’s documentation.

License: Apache License 2.0

Any restrictions to use by non-academics: none.

## Supplementary Information

**Additional file 1. **Jupyter notebook (html export) containing the analysis detailed in the manuscript. The code and the dataset are also available in the in the GitHub repository, https://github.com/chemosim-lab/ProLIF-paper, or through the Zenodo archive, https://doi.org/10.5281/zenodo.4945869.

## Data Availability

The datasets and code supporting the conclusions of this article are available in the supplementary materials and in the GitHub repository, https://github.com/chemosim-lab/ProLIF-paper, or through the Zenodo archive, https://doi.org/10.5281/zenodo.4945869.
