# Supplementary material for: ProLIF: a library to encode molecular interactions as fingerprints
Source: J Cheminform. 2021 Sep 25;13:72. doi: 10.1186/s13321-021-00548-6 (PMC8466659; doi:10.1186/s13321-021-00548-6)
Supplement: Supplementary file 1 — Additional file 1. Jupyter notebook (html export) containing the analysis detailed in the manuscript. The code and the dataset are also available in the in the GitHub repository, https://github.com/chemosim-lab/ProLIF-paper, or through the Zenodo archive, https://doi.org/10.5281/zenodo.4945869. [file 13321_2021_548_MOESM1_ESM.html]

Analysis


In [1]:

```
import itertools
from functools import lru_cache
from collections import defaultdict
import json
import re
from html import escape
import warnings

import pandas as pd
import numpy as np
from matplotlib import pyplot as plt
from matplotlib.patches import Patch, Rectangle
import seaborn as sns
from IPython.display import IFrame, HTML
from sklearn.cluster import KMeans
from scipy.cluster.vq import vq
import networkx as nx
from pyvis.network import Network
from rdkit import Chem
from rdkit.Chem import DataStructs
import MDAnalysis as mda
from MDAnalysis.topology.tables import vdwradii
import prolif as plf
from prolif.plotting.network import LigNetwork

warnings.filterwarnings("ignore", "Consider using IPython.display.IFrame instead")
```

## 1. MD trajectory of a GPCR in complex with a ligand¶

---

The trajectory of 5-HT1B receptor in complex with ergotamine was downloaded from GPCRmd, id 90:

- Topology
- Trajectory

There are 1.5 µs of total simulation time (3 replicates), with .2 ns between each frame, but we'll only analyze one replicate here (500ns).

The trajectory is already centered in the box so no need to postprocess it.

In [2]:

```
# download MD simulation files if necessary
data = pathlib.Path("../Data")
url = "https://submission.gpcrmd.org/dynadb/files/Dynamics"
for filename in ["10872_dyn_90.psf", "10869_trj_90.xtc"]:
    path = data / filename
    if not path.is_file():
        target = f"{url}/{filename}"
        !wget {target} -P {str(data)} --quiet --show-progress

# load trajectory with MDAnalysis
u = mda.Universe(
    "../Data/10872_dyn_90.psf",
    "../Data/10869_trj_90.xtc"
)
elements = [mda.topology.guessers.guess_atom_element(n) for n in u.atoms.names]
u.add_TopologyAttr("elements", elements)
# create selections for the ligand and protein
lig = u.atoms.select_atoms("resname ERM")
prot = u.atoms.select_atoms("protein")
# use default interactions
fp = plf.Fingerprint()
```

In [3]:

```
# run on a slice of frames from begining to end with a step of 5
# so that there's 1ns between each analyzed frames
fp.run(u.trajectory[::5], lig, prot)
# convert the results to a pandas DataFrame
df = fp.to_dataframe()
df.to_pickle("pickles/ifp_df.pkl")
# show only the 5 first frames
df.head(5)
```

```

```

Out[3]:

| ligand | ERM1 | | | | | | | | | | | | | | | | | | | | |
| --- | --- | --- | --- | --- | --- | --- | --- | --- | --- | --- | --- | --- | --- | --- | --- | --- | --- | --- | --- | --- | --- |
| protein | TYR38 | ILE39 | TYR40 | SER106 | TYR109 | THR110 | TRP125 | LEU126 | ASP129 | | ... | PHE346 | | LEU348 | PHE351 | | ASP352 | THR355 | | TRP356 | TYR359 |
| interaction | Hydrophobic | Hydrophobic | Hydrophobic | Hydrophobic | Hydrophobic | Hydrophobic | Hydrophobic | Hydrophobic | Hydrophobic | HBDonor | ... | Hydrophobic | PiStacking | Hydrophobic | Hydrophobic | PiStacking | Hydrophobic | Hydrophobic | HBAcceptor | Hydrophobic | Hydrophobic |
| Frame |  |  |  |  |  |  |  |  |  |  |  |  |  |  |  |  |  |  |  |  |  |
| 0 | False | False | False | False | True | False | True | True | True | True | ... | False | False | False | True | False | True | True | False | False | True |
| 5 | False | False | False | False | True | False | True | True | True | True | ... | False | False | False | True | False | True | True | False | False | True |
| 10 | False | False | False | False | True | False | True | True | True | True | ... | False | False | False | True | False | True | True | False | False | True |
| 15 | False | False | False | False | True | False | True | False | True | True | ... | False | False | False | True | False | True | True | False | False | True |
| 20 | False | False | False | False | True | False | True | True | True | True | ... | False | False | False | True | False | True | True | False | False | True |

5 rows × 50 columns

In [4]:

```
# with atom indices
df = fp.to_dataframe(return_atoms=True)
df.to_pickle("pickles/ifp_df_atoms.pkl")
```

### 1.a) Data analysis with pandas¶

In [5]:

```
df = (pd.read_pickle("pickles/ifp_df.pkl")
        .astype(np.uint8))
```

In [6]:

```
# ignore an interaction
(df.drop("Hydrophobic", level="interaction", axis=1)
   .head(5))
```

Out[6]:

| ligand | ERM1 | | | | | | | | | | | | |
| --- | --- | --- | --- | --- | --- | --- | --- | --- | --- | --- | --- | --- | --- |
| protein | ASP129 | | THR134 | VAL201 | SER212 | THR213 | PHE330 | | PHE331 | TRP345 | PHE346 | PHE351 | THR355 |
| interaction | HBDonor | Cationic | HBDonor | HBAcceptor | HBDonor | HBDonor | CationPi | PiStacking | PiStacking | HBAcceptor | PiStacking | PiStacking | HBAcceptor |
| Frame |  |  |  |  |  |  |  |  |  |  |  |  |  |
| 0 | 1 | 1 | 0 | 1 | 1 | 0 | 0 | 0 | 1 | 0 | 0 | 0 | 0 |
| 5 | 1 | 1 | 0 | 1 | 0 | 0 | 0 | 0 | 1 | 0 | 0 | 0 | 0 |
| 10 | 1 | 1 | 0 | 1 | 0 | 0 | 0 | 1 | 1 | 0 | 0 | 0 | 0 |
| 15 | 1 | 1 | 0 | 1 | 0 | 0 | 0 | 0 | 0 | 0 | 0 | 0 | 0 |
| 20 | 1 | 1 | 0 | 1 | 0 | 0 | 0 | 1 | 1 | 0 | 0 | 0 | 0 |

In [7]:

```
# share, by residue, of pi-stacking interactions
g = (df.xs("PiStacking", level="interaction", axis=1)
       .sum())
(100 * g / g.sum()).sort_values(ascending=False)
```

Out[7]:

```
ligand  protein
ERM1    PHE331     48.368794
        PHE330     28.652482
        PHE351     22.269504
        PHE346      0.709220
dtype: float64
```

F331 makes half of the PiStacking interactions

In [8]:

```
# share, by interaction type, of SER212 interactions
g = (df.xs("SER212", level="protein", axis=1)
       .sum())
(100 * g / g.sum()).sort_values(ascending=False)
```

Out[8]:

```
ligand  interaction
ERM1    Hydrophobic    68.567455
        HBDonor        31.432545
dtype: float64
```

1/3 of S212 interactions are as an H-bond donor

In [9]:

```
# occurence percentage of each interaction along the simulation
(df.groupby(level="interaction", axis=1)
   .sum()
   .astype(bool)
   .mean()
   .sort_values(ascending=False) * 100)
```

Out[9]:

```
interaction
Hydrophobic    100.0
HBDonor        100.0
Cationic       100.0
HBAcceptor      91.8
PiStacking      85.0
CationPi         1.6
dtype: float64
```

There are hydrophobic, H-bond donor, and cationic interactions in all frames

In [10]:

```
# share of each interaction averaged on all frames
g = (df.groupby(level="interaction", axis=1)
       .sum()
       .mean())
(100 * g / g.sum()).sort_values(ascending=False)
```

Out[10]:

```
interaction
Hydrophobic    80.808402
HBDonor         5.792489
PiStacking      5.609484
Cationic        3.978358
HBAcceptor      3.747613
CationPi        0.063654
dtype: float64
```

On average, in each frame 80% of interactions are hydrophobic, 6% are H-bond donor, 6% pi-stacking...etc.

In [11]:

```
# 10 residues most frequently interacting with the ligand
(df.groupby(level=["ligand", "protein"], axis=1)
   .sum()
   .astype(bool)
   .mean()
   .sort_values(ascending=False)
   .head(10))
```

Out[11]:

```
ligand  protein
ERM1    ASP129     1.000
        ILE130     1.000
        PHE330     1.000
        VAL201     0.998
        PHE331     0.998
        SER212     0.988
        TRP327     0.982
        VAL200     0.982
        CYS133     0.976
        PHE351     0.974
dtype: float64
```

### 1.b) Similarity between binding modes¶

In [12]:

```
# compute tanimoto similarity matrix
bvs = plf.to_bitvectors(df)
mat = []
for bv in bvs:
    mat.append(DataStructs.BulkTanimotoSimilarity(bv, bvs))
mat = pd.DataFrame(mat, index=df.index, columns=df.index)
```

In [13]:

```
# display heatmap
fig, ax = plt.subplots(figsize=(3, 3), dpi=200)
sns.heatmap(
    mat, ax=ax, square=True,
    cmap="icefire",
    vmin=.3, vmax=1, center=.7,
    xticklabels=75, yticklabels=75,
)

ax.invert_yaxis()
fig.patch.set_facecolor('white')
```

In [14]:

```
fig.savefig("../Figures/similarity_matrix.png", bbox_inches="tight", dpi=300)
```

We can also regroup frames of the same clusters with KMeans

In [15]:

```
# KMeans clustering

def bv_to_np(bv):
    """convert rdkit bitvector to numpy array"""
    arr = np.zeros((0,))
    DataStructs.ConvertToNumpyArray(bv, arr)
    return arr

fps = np.array([bv_to_np(bv) for bv in bvs])
kmean = KMeans(n_clusters=2, random_state=0)
clusters = kmean.fit_predict(fps)
```

In [16]:

```
# display heatmap sorted by cluster
fig, ax = plt.subplots(figsize=(3, 3), dpi=200)
sns.heatmap(
    # matrix sorted by cluster
    (mat
     .set_axis(clusters, axis=0)
     .set_axis(clusters, axis=1)
     .sort_index(axis=0)
     .sort_index(axis=1)
    ),
    ax=ax, square=True,
    cmap="icefire",
    vmin=.3, vmax=1, center=.7,
    xticklabels=75,
    yticklabels=0,
)

# show tick on heatmap's y-axis when new cluster
sc = np.sort(clusters)
# find index where a new cluster is assigned
s = np.argwhere(sc[1:] - sc[:-1]) + 1
s = s.flatten().tolist()
s = [0] + s + [sc.size]
# set on y axis
ax.set_yticks(s)
ax.set_xticks(s)
ax.invert_yaxis()
# ax.yaxis.labelpad = 8
for c, (i1, i2) in enumerate(zip(s[:-1], s[1:])):
    ax.text(-42, (i1 + i2) / 2, c, rotation=90)
    ax.text((i1 + i2) / 2, -42, c, rotation=0)
fig.patch.set_facecolor('white')
```

In [17]:

```
fig.savefig("../Figures/similarity_matrix_clustered.png", bbox_inches="tight", dpi=300)
```

In [18]:

```
# get closest frame to each cluster center
closest, dists = vq(kmean.cluster_centers_, fps)
data = df.iloc[closest].copy()
# only show columns with differences between clusters
data.loc[:, data.nunique() > 1]
```

Out[18]:

| ligand | ERM1 | | | | | | |
| --- | --- | --- | --- | --- | --- | --- | --- |
| protein | TRP125 | THR203 | THR209 | SER212 | SER334 | PHE351 | ASP352 |
| interaction | Hydrophobic | Hydrophobic | Hydrophobic | HBDonor | Hydrophobic | PiStacking | Hydrophobic |
| Frame |  |  |  |  |  |  |  |
| 420 | 1 | 0 | 0 | 1 | 0 | 1 | 0 |
| 1680 | 0 | 1 | 1 | 0 | 1 | 0 | 1 |

### 1.c) Display LigPlot¶

We'll aggregate the IFP and only display interactions that occur in at least 30% of frames

In [19]:

```
df = pd.read_pickle("pickles/ifp_df_atoms.pkl")
lmol = plf.Molecule.from_mda(lig)
net = LigNetwork.from_ifp(df, lmol, kind="aggregate", threshold=.3, rotation=110)
net.show("../Figures/lignetwork.html")
```

Out[19]:

## 2. Protein-protein interactions (PPI) within the GPCR¶

---

Inactive vs active forms of the bovine rhodopsin. PQR file were prepared by submitting a job to the PDB2PQR webserver with:

- PDB ids 1U19 (inactive form) and 6FK6 (active form)
- AMBER force-field and naming scheme
- PROPKA to assign protonation states at pH 7.0
- Keeping existing chain IDs
- Dropping water molecules

In [20]:

```
fp = plf.Fingerprint()
```

#### Inactive form (1U19)¶

In [21]:

```
# use RDKit to read PQR file
mol = Chem.MolFromPDBFile("../Data/1u19.pqr", removeHs=False)
# convert to MDAnalysis
u = mda.Universe(mol)
gpcr = u.select_atoms("protein and chainID A")
fp.run(u.trajectory, gpcr, gpcr)
inactive = fp.to_dataframe()
inactive
```

```

```

Out[21]:

| ligand | ACE0.A | | MET1.A | | | | ASN2.A | | | | ... | VAL345.A | ALA346.A | | | PRO347.A | | | | ALA348.A | |
| --- | --- | --- | --- | --- | --- | --- | --- | --- | --- | --- | --- | --- | --- | --- | --- | --- | --- | --- | --- | --- | --- |
| protein | ACE0.A | MET1.A | ACE0.A | MET1.A | ASN2.A | GLY3.A | MET1.A | ASN2.A | GLY3.A | GLY280.A | ... | PRO347.A | VAL345.A | ALA346.A | PRO347.A | VAL345.A | ALA346.A | PRO347.A | ALA348.A | PRO347.A | ALA348.A |
| interaction | Hydrophobic | Hydrophobic | Hydrophobic | Hydrophobic | Hydrophobic | Hydrophobic | Hydrophobic | Hydrophobic | Hydrophobic | Hydrophobic | ... | Hydrophobic | Hydrophobic | Hydrophobic | Hydrophobic | Hydrophobic | Hydrophobic | Hydrophobic | Hydrophobic | Hydrophobic | Hydrophobic |
| Frame |  |  |  |  |  |  |  |  |  |  |  |  |  |  |  |  |  |  |  |  |  |
| 0 | True | True | True | True | True | True | True | True | True | True | ... | True | True | True | True | True | True | True | True | True | True |

1 rows × 3585 columns

#### Active form (6FK6)¶

In [22]:

```
# use RDKit to read PQR file
mol = Chem.MolFromPDBFile("../Data/6fk6.pqr", removeHs=False)
# convert to MDAnalysis
u = mda.Universe(mol)
gpcr = u.select_atoms("protein")
fp.run(u.trajectory, gpcr, gpcr)
active = fp.to_dataframe()
active
```

```

```

Out[22]:

| ligand | ACE0.A | | | MET1.A | | | CYX2.A | | | | ... | GLY324.A | | LYS325.A | | | | | ASN326.A | | |
| --- | --- | --- | --- | --- | --- | --- | --- | --- | --- | --- | --- | --- | --- | --- | --- | --- | --- | --- | --- | --- | --- |
| protein | ACE0.A | MET1.A | CYX2.A | ACE0.A | MET1.A | CYX2.A | ACE0.A | MET1.A | CYX2.A | GLY3.A | ... | GLY324.A | LYS325.A | CYS322.A | | GLY324.A | LYS325.A | ASN326.A | VAL318.A | LYS325.A | ASN326.A |
| interaction | Hydrophobic | Hydrophobic | Hydrophobic | Hydrophobic | Hydrophobic | Hydrophobic | Hydrophobic | Hydrophobic | Hydrophobic | Hydrophobic | ... | Hydrophobic | Hydrophobic | Hydrophobic | HBDonor | Hydrophobic | Hydrophobic | Hydrophobic | Hydrophobic | Hydrophobic | Hydrophobic |
| Frame |  |  |  |  |  |  |  |  |  |  |  |  |  |  |  |  |  |  |  |  |  |
| 0 | True | True | True | True | True | True | True | True | True | True | ... | True | True | True | True | True | True | True | True | True | True |

1 rows × 3277 columns

In [23]:

```
df = (
    pd.concat([
        inactive.set_axis(["Inactive"], axis=0),
        active.set_axis(["Active"], axis=0),
    ]).fillna(False)
)
df.to_pickle("pickles/ppi_state.pkl")
df
```

Out[23]:

| ligand | ACE0.A | | | ALA117.A | | | | | | | ... | VAL87.A | | | | | | | | | |
| --- | --- | --- | --- | --- | --- | --- | --- | --- | --- | --- | --- | --- | --- | --- | --- | --- | --- | --- | --- | --- | --- |
| protein | ACE0.A | CYX2.A | MET1.A | ALA117.A | GLH113.A | | GLU113.A | GLY120.A | GLY121.A | GLY89.A | ... | GLY51.A | ILE48.A | LEU84.A | MET86.A | PHE52.A | PHE88.A | PHE91.A | THR92.A | | VAL87.A |
| interaction | Hydrophobic | Hydrophobic | Hydrophobic | Hydrophobic | HBDonor | Hydrophobic | HBDonor | Hydrophobic | HBAcceptor | Hydrophobic | ... | Hydrophobic | Hydrophobic | Hydrophobic | Hydrophobic | Hydrophobic | Hydrophobic | Hydrophobic | HBAcceptor | Hydrophobic | Hydrophobic |
| Inactive | True | False | True | True | False | False | True | True | True | False | ... | True | True | True | True | True | True | True | False | True | True |
| Active | True | True | True | True | True | True | False | False | True | True | ... | True | False | True | True | True | True | True | True | False | True |

2 rows × 4210 columns

In [24]:

```
# read annotated sequence from GPCRdb (bovine opsd)
opsd = pd.read_excel("../Data/opsd_gpcrdb.xlsx",
                      header=None, skipfooter=2)
opsd.values[0, 0] = "Label"
opsd.values[1, 0] = "Helix number"
opsd.values[-1, 0] = "Sequence"
opsd.set_index(0, inplace=True)
opsd.iloc[0].fillna(method="ffill", inplace=True)
opsd = opsd.T

@lru_cache(maxsize=None)
def get_opsd_info(number):
    return opsd.iloc[number - 1]

opsd
```

Out[24]:

|  | Label | Helix number | Sequence-based (BW) | Structure-based (GPCRdb) | Sequence |
| --- | --- | --- | --- | --- | --- |
| 1 | N-term | NaN | NaN | NaN | M |
| 2 | N-term | NaN | NaN | NaN | N |
| 3 | N-term | NaN | NaN | NaN | G |
| 4 | N-term | NaN | NaN | NaN | T |
| 5 | N-term | NaN | NaN | NaN | E |
| ... | ... | ... | ... | ... | ... |
| 344 | C-term | NaN | NaN | NaN | Q |
| 345 | C-term | NaN | NaN | NaN | V |
| 346 | C-term | NaN | NaN | NaN | A |
| 347 | C-term | NaN | NaN | NaN | P |
| 348 | C-term | NaN | NaN | NaN | A |

348 rows × 5 columns

In [25]:

```
# drop some of the interactions
df = pd.read_pickle("pickles/ppi_state.pkl")
df.drop("Hydrophobic", axis=1, level="interaction", inplace=True)
mask = [True for _ in df.columns]
for i, (l, p, interaction) in enumerate(df.columns):
    if mask[i] == False:
        continue
    lr = plf.ResidueId.from_string(l)
    pr = plf.ResidueId.from_string(p)
    li = get_opsd_info(lr.number)["Label"]
    pi = get_opsd_info(pr.number)["Label"]
    same_domain = li == pi
    l_is_helix = li.startswith("TM") or li.startswith("H")
    p_is_helix = pi.startswith("TM") or pi.startswith("H")
    same_helix = same_domain and l_is_helix
    if (
        (abs(pr.number - lr.number) < 3) # filter out covalent and neighbor in sequence
        or same_helix # filter out interactions within same helix
        or not (l_is_helix and p_is_helix) # filter out if one of the residues isn't in a helix
    ):
        mask[i] = False
    # remove symetrical interaction
    try:
        col = df.columns.get_loc((p, l, interaction))
    except KeyError:
        pass
    else:
        mask[col] = False
    
df = df[df.columns[mask]]
df.shape
```

Out[25]:

```
(2, 47)
```

In [26]:

```
# differences between active and inactive
df.loc[:, df.nunique() > 1]
```

Out[26]:

| ligand | ALA132.A | ALA234.A | ARG135.A | | | | ARG314.A | ASN73.A | CYS140.A | CYS222.A | ... | PHE221.A | PHE293.A | PHE313.A | SER240.A | THR229.A | THR251.A | THR320.A | TRP265.A | TYR223.A | TYR306.A |
| --- | --- | --- | --- | --- | --- | --- | --- | --- | --- | --- | --- | --- | --- | --- | --- | --- | --- | --- | --- | --- | --- |
| protein | CYS222.A | GLN244.A | GLU247.A | | THR251.A | TYR223.A | ILE307.A | TYR306.A | THR229.A | ALA132.A | ... | TYR136.A | TYR43.A | TYR306.A | GLN237.A | CYS140.A | ARG135.A | GLN64.A | PHE212.A | ARG135.A | ASN73.A |
| interaction | HBAcceptor | HBAcceptor | Cationic | HBDonor | HBDonor | HBDonor | HBDonor | HBDonor | HBDonor | HBDonor | ... | PiStacking | PiStacking | PiStacking | HBDonor | HBAcceptor | HBAcceptor | HBDonor | HBDonor | HBAcceptor | HBAcceptor |
| Inactive | True | False | True | True | True | False | True | True | False | True | ... | False | False | True | False | False | True | False | False | False | True |
| Active | False | True | False | False | False | True | False | False | True | False | ... | True | True | False | True | True | False | True | True | True | False |

2 rows × 33 columns

In [27]:

```
# group different interactions types together and assign:
# 1 if only in active, -1 if only in inactive, 0 if in both
data = (
    df
    .groupby(level=["ligand", "protein"], axis=1, sort=False)
    .sum()
    .astype(bool)
    .astype(int)
    .diff()
    .iloc[-1]
)

colors = {
    "TM1": "#ccebc5",
    "TM2": "#ffffb3",
    "TM3": "#bebada",
    "TM4": "#fb8072",
    "TM5": "#80b1d3",
    "TM6": "#bc80bd",
    "TM7": "#b3de69",
    "H8": "#fccde5",
}

G = nx.Graph()
# add nodes
residues = sorted(np.unique([plf.ResidueId.from_string(r)
                             for i in data.index.values
                             for r in i]))

# skip if residue is different (mutated) between structures
temp = defaultdict(list)
for resid in residues:
    temp[resid.number].append(str(resid))

for resid in residues:
    if len(temp[resid.number]) > 1:
        print(resid.number)
        continue
    res = str(resid)
    resid = plf.ResidueId.from_string(res)
    info = get_opsd_info(resid.number)
    name = f'{info["Sequence"]}{resid.number}'
    domain = info["Label"]
    color = colors.get(domain, "#cccccc")
    if color == "#cccccc":
        continue
    hnum = info["Helix number"]
    if isinstance(hnum, str):
        note = (f'{hnum}.{info["Sequence-based (BW)"]}'
                f'x{info["Structure-based (GPCRdb)"]}')
        title = note
    else:
        title = domain
    title = f"{name}<br/>{title}"
    G.add_node(res, label=f"{name:^4s}", title=title, borderWidth=1,
               shape="circle", color=color, resid=res)

# add interactions
for resids, value in data.items():
    label = "{} - {}<br/>{}".format(*resids, "<br/>".join([f"{k}: {v}"
                                   for k, v in (df.xs(resids,
                                                      level=["ligand", "protein"],
                                                      axis=1)
                                                  .sum()
                                                  .to_dict()
                                                  .items())]))
    width = 15
    if value == 1:
        color = "#66c2a5"
    elif value == -1:
        color = "#fc8d62"
    else:
        color = "#a9a9a9"
    info = {get_opsd_info(plf.ResidueId.from_string(r).number)["Label"] for r in resids}
    if "TM7" in info:
        if "H8" in info:
            smooth = {"type": "curvedCCW"}
        else:
            smooth = {"type": "curvedCW"}
    elif ("TM1" in info) and ("H8" in info):
        smooth = {"type": "curvedCW"}
    else:
        smooth = {"type": "curvedCCW"}
    G.add_edge(
        *resids, title=label, data=value,
        color=color, width=width, smooth=smooth,
    )

# scale node by number of state-dependent interactions 
for res, adj in G.adjacency():
    n = sum([d["data"] != 10 for d in adj.values()])
    G.nodes[res]["font"] = {
        "face": "Monospace",
        "size": 50+(n+2)**3,
    }

# convert to pyvis network
net = Network(width=1000, height=1000,  notebook=True, heading="")
net.from_nx(G)

# sort nodes by fontsize so that bigger nodes are drawn on top
net.nodes = sorted(net.nodes, key=lambda x: x["font"]["size"])

# force circular layout
layout = nx.circular_layout(G)
for node in net.nodes:
    node["x"] = layout[node["id"]][0] * 2000
    node["y"] = layout[node["id"]][1] * 2000
net.toggle_physics(False)

net.write_html("../Figures/ppi_state.html")
IFrame("../Figures/ppi_state.html", width="100%", height=800)
```

Out[27]:

In [28]:

```
# legend for the corresponding figure in the paper
fig, ax = plt.subplots(figsize=(1, 2), dpi=200)
handles = []
for domain, hexcol in colors.items():
    item = Patch(color=hexcol, label=domain)
    handles.append(item)
leg = plt.legend(handles=handles, ncol=1, loc="upper left")
plt.axis('off')
fig.savefig("../Figures/gpcr_ppi_legend.png", bbox_inches="tight", dpi=300)
```

## 3. Protein-protein interactions between a GPCR and a G protein¶

Analysis of PPI for the β2 adrenoreceptor (PDB 3SN6) which contains a class A GPCR in complex if a G protein

PQR file was prepared by submitting a job to the PDB2PQR webserver with:

- PDB id 3SN6
- AMBER force-field and naming scheme
- PROPKA to assign protonation states at pH 7.0
- Keeping existing chain IDs
- Dropping water molecules

In [29]:

```
# use RDKit to read PQR file
mol = Chem.MolFromPDBFile("../Data/3sn6.pqr", removeHs=False)
# convert to MDAnalysis
u = mda.Universe(mol)
gpcr = u.select_atoms("protein and chainID R")
gprotein = u.select_atoms("protein and not chainID R")
gpcr, gprotein
```

Out[29]:

```
(<AtomGroup with 7175 atoms>, <AtomGroup with 13670 atoms>)
```

Custom implementation of the van der Waals contact interaction used in the original paper by Flock et al.

In [30]:

```
class VdWContact(plf.interactions.Interaction):
    """Interaction based on the van der Waals radii of interacting atoms.
    
    Parameters
    ----------
    tolerance : float
        Tolerance added to the sum of vdW radii of atoms before comparing to
        the interatomic distance. If ``distance <= sum_vdw + tolerance`` the
        atoms are identified as a contact
    """
    def __init__(self, tolerance=.6):
        self.tolerance = tolerance

    def detect(self, ligand, residue):
        lxyz = ligand.GetConformer()
        rxyz = residue.GetConformer()
        for la, ra in itertools.product(ligand.GetAtoms(), residue.GetAtoms()):
            lig_atom = la.GetSymbol().upper()
            res_atom = ra.GetSymbol().upper()
            sum_vdw = vdwradii[lig_atom] + vdwradii[res_atom]
            dist = (lxyz.GetAtomPosition(la.GetIdx())
                        .Distance(rxyz.GetAtomPosition(ra.GetIdx())))
            if dist - sum_vdw <= self.tolerance:
                return True, la.GetIdx(), ra.GetIdx()
        return False, None, None
```

In [31]:

```
# use custom vdw contact class in prolif
# and run on the multiple complexes (GPCR/GPCR, Gprot/Gprot, and Gprot/GPCR)
fp = plf.Fingerprint(["VdWContact"])
fp.run(u.trajectory, gprotein, gpcr)
df_i = fp.to_dataframe()
fp.run(u.trajectory, gprotein, gprotein)
df_gprot = fp.to_dataframe()
fp.run(u.trajectory, gpcr, gpcr)
df_gpcr = fp.to_dataframe()
```

```
/home/cedric/projects/mdanalysis/package/MDAnalysis/converters/RDKit.py:975: UserWarning: The standardization could not be completed within a reasonable number of iterations
  warnings.warn("The standardization could not be completed within a "
```

```

```

```

```

```

```

In [32]:

```
# read annotated sequences from GPCRdb
adrb2 = pd.read_excel("../Data/adrb2_gpcrdb.xlsx",
                      header=None, skipfooter=2)
adrb2.values[0, 0] = "Label"
adrb2.values[1, 0] = "Helix number"
adrb2.values[-1, 0] = "Sequence"
adrb2.set_index(0, inplace=True)
adrb2.iloc[0].fillna(method="ffill", inplace=True)
adrb2 = adrb2.T

@lru_cache(maxsize=None)
def get_adrb2_info(number):
    # in the PDB input file, endolysin is numbered starting from 1000
    # but we don't need it here
    if number >= 1000:
        return {"Label": "nan"}
    return adrb2.iloc[number - 1]

gnas = pd.read_excel("../Data/gnas_gpcrdb.xlsx",
                      header=None, skipfooter=2)
gnas.values[0, 0] = "Label"
gnas.values[1, 0] = "Helix number"
gnas.values[-1, 0] = "Sequence"
gnas.set_index(0, inplace=True)
gnas.iloc[0].fillna(method="ffill", inplace=True)
gnas = gnas.T

@lru_cache(maxsize=None)
def get_gnas_info(number):
    return gnas.iloc[number - 1]

adrb2
```

Out[32]:

|  | Label | Helix number | Sequence-based (BW) | Structure-based (GPCRdb) | Sequence |
| --- | --- | --- | --- | --- | --- |
| 1 | N-term | NaN | NaN | NaN | M |
| 2 | N-term | NaN | NaN | NaN | G |
| 3 | N-term | NaN | NaN | NaN | Q |
| 4 | N-term | NaN | NaN | NaN | P |
| 5 | N-term | NaN | NaN | NaN | G |
| ... | ... | ... | ... | ... | ... |
| 409 | C-term | NaN | NaN | NaN | N |
| 410 | C-term | NaN | NaN | NaN | D |
| 411 | C-term | NaN | NaN | NaN | S |
| 412 | C-term | NaN | NaN | NaN | L |
| 413 | C-term | NaN | NaN | NaN | L |

413 rows × 5 columns

In [33]:

```
# filter out some of the interactions
def filter_interactions(df, contacts, gpcr=False):
    mask = []
    for l, p, interaction in df.columns:
        lr = plf.ResidueId.from_string(l)
        pr = plf.ResidueId.from_string(p)
        get_info = get_adrb2_info if gpcr else get_gnas_info
        same_helix = get_info(lr.number)["Label"] == get_info(pr.number)["Label"]
        if same_helix:
            if gpcr:
                same_helix &= get_info(lr.number)["Label"].startswith("TM")
            else:
                same_helix &= get_info(lr.number)["Label"].startswith("H")
        if (
            (abs(pr.number - lr.number) <= 1) or # filter out covalent
            (same_helix ) or # filter out interactions within same helix
            any(r not in contacts for r in (l, p)) or # filter out residues that don't appear in gpcr-prot interactions
            (gpcr and any(r.number >= 1000 for r in (lr, pr))) # filter out endolysin
           ):
            mask.append(False)
        else:
            mask.append(True)
    return df[df.columns[mask]]

def sort_ifp_columns(df, order=["ligand", "protein", "interaction"]):
    df = df.copy()
    cols = np.array([(
            plf.ResidueId.from_string(i[0]),
            plf.ResidueId.from_string(i[1]),
            i[2])
        for i in df.columns.values],
        dtype=[("ligand", object), ("protein", object), ("interaction", object)])
    df.columns = df.columns[np.argsort(cols, order=order)]
    df = df.reorder_levels(order, axis=1)
    return df

df = df_i.copy()
contacts = np.unique([list(i[:2]) for i in df_i.columns.values])
d = filter_interactions(df_gprot, contacts)
df = pd.concat([df, d], axis=1)
d = filter_interactions(df_gpcr, contacts, gpcr=True)
df = pd.concat([df, d], axis=1)
df = sort_ifp_columns(df)
df = df.droplevel("interaction", axis=1)
df.to_pickle("pickles/ppi.pkl")
```

In [34]:

```
df = pd.read_pickle("pickles/ppi.pkl")
contacts = np.array([list(i) for i in df.columns.values])
contacts = np.unique(np.sort(contacts, axis=1), axis=0)
len(contacts)
```

Out[34]:

```
64
```

In [35]:

```
# custom vis.js network interface, because pyvis implementation
# hasn't been updated in a while and doesn't support some of the
# arguments needed to make this figure
class Network:
    _JS_TEMPLATE = """
        var nodes, edges;
        function drawGraph(_id, nodes, edges, options) {
            var container = document.getElementById(_id);
            nodes = new vis.DataSet(nodes);
            edges = new vis.DataSet(edges);
            var data = {nodes: nodes, edges: edges};
            var network = new vis.Network(container, data, options);
            %(post_initialization)s
            return network;
        }
        nodes = %(nodes)s;
        edges = %(edges)s;
        network = drawGraph('%(div_id)s', nodes, edges, %(options)s);
    """
    _HTML_TEMPLATE = """
        <html>
        <head>
        <script type="text/javascript" src="https://unpkg.com/vis-network@9.0.4/dist/vis-network.min.js"></script>
        <link href="https://unpkg.com/vis-network@9.0.4/dist/dist/vis-network.min.css" rel="stylesheet" type="text/css" />
        <style type="text/css">
            body { padding: 0; margin: 0; }
        </style>
        </head>
        <body>
        <div id="mynetwork"></div>
        <div id="networklegend"></div>
        <script type="text/javascript">
            %(js)s
        </script>
        </body>
        </html>
    """
    def __init__(self):
        self.nodes = []
        self.edges = []
        self.post_initialization = ""

    def add_node(self, _id, **kwargs):
        self.nodes.append({"id": _id, **kwargs})

    def add_edge(self, _from, to, **kwargs):
        self.edges.append({"from": _from, "to": to, **kwargs})
    
    def _get_js(self, width="100%", height="500px", div_id="mynetwork",
        fontsize=20):
        """Returns the JavaScript code to draw the network"""
        self.width = width
        self.height = height
        options = {
            "width": width,
            "height": height,
        }
        options.update(self.options)
        js = self._JS_TEMPLATE % dict(div_id=div_id,
                                      nodes=json.dumps(self.nodes),
                                      edges=json.dumps(self.edges),
                                      options=json.dumps(options),
                                      post_initialization=self.post_initialization,
                                     )
        return js
    
    def _get_html(self, **kwargs):
        """Returns the HTML code to draw the network"""
        return self._HTML_TEMPLATE % dict(js=self._get_js(**kwargs))

    def display(self, **kwargs):
        """Prepare and display the network"""
        html = self._get_html(**kwargs)
        iframe = ('<iframe width="{width}" height="{height}" frameborder="0" '
                  'srcdoc="{doc}"></iframe>')
        return HTML(iframe.format(width=self.width, height=self.height,
                                  doc=escape(html)))
    
    def show(self, filename, **kwargs):
        """Save and display the network"""
        html = self._get_html(**kwargs)
        with open(filename, "w") as f:
            f.write(html)
        iframe = ('<iframe width="{width}" height="{height}" frameborder="0" '
                  'src="{filename}"></iframe>')
        return HTML(iframe.format(width=self.width, height="500px" if "%" in self.height else self.height,
                                  filename=filename))
```

In [36]:

```
# plot the network

net = Network()
colors = {
    "ICL1": "#f7fcfd",
    "TM3": "#e5f5f9",
    "ICL2": "#ccece6",
    "TM5": "#99d8c9",
    "ICL3": "#66c2a4",
    "TM6": "#41ae76",
    
    "hns1": "#fff7fb",
    "S1": "#ece7f2",
    "S3": "#d0d1e6",
    "H4": "#a6bddb",
    "h4s6": "#74a9cf",
    "H5": "#3690c0",
}
rid_cache = {}
nodes = np.unique(contacts)

for r in nodes:
    rid = plf.ResidueId.from_string(r)
    rid_cache[r] = rid
    label = f"{rid.name}{rid.number}"
    if rid.chain == "R":
        info = get_adrb2_info(rid.number)
        struct = info["Label"]
        hnum = info["Helix number"]
        if isinstance(hnum, str):
            note = (f'{hnum}.{info["Sequence-based (BW)"]}'
                    f'x{info["Structure-based (GPCRdb)"]}')
            title = f"{struct}\n{note}"
            label = f"{note}\n{label}"
        else:
            title = struct
            label = f"{struct}\n{label}"
        color = colors.get(struct, "grey")
        net.add_node(r, label=label, title=title, shape="box",
                     color=color, dtype="gpcr", margin=20)
    else:
        if rid.chain == "A":
            info = get_gnas_info(rid.number)
            struct = info["Label"]
            if isinstance(info["Helix number"], str):
                note = (f'{info["Helix number"]}.{info["Sequence-based ()"]}'
                        f'.{info["Structure-based (GPCRdb)"]}')
                title = f"Gs-α\n{note}"
                label = f"{note}\n{label}"
            else:
                title = "Gαs"
            color = colors.get(struct, "grey")
            net.add_node(r, label=label, title=title, shape="circle",
                         color=color, dtype="gprotein")
        else:
            title = "Gβ1"
            label = f"{title}\n{label}"
            net.add_node(r, label=label, title=title, shape="circle",
                         color="#fec44f", dtype="gprotein")

for r1, r2 in contacts:
    rid1 = rid_cache[r1]
    rid2 = rid_cache[r2]
    label = f"{r1} - {r2}"
    same_chain = rid1.chain == rid2.chain
    width = 3
    length = 100 if same_chain else 300
    dashes = [10] if same_chain else False
    net.add_edge(r1, r2, length=length, title=label, color="#a9a9a9",
                 width=width, selectionWidth=6, dashes=dashes)

for i in range(len(nodes)):
    node = net.nodes[i]
    nid = node["id"]
    n_neighbors = 0
    for edge in net.edges:
        if nid in [edge["from"], edge["to"]]:
            n_neighbors += 1
    node["margin"] = max(5, n_neighbors*3.5)

net.options = {
    "nodes": {
        "font": {
            "size": 36,
        },
    },
    "edges": {
        "smooth": {
            "type": "continuous",
        },
    },
    "physics": {
        "hierarchicalRepulsion": {
            "avoidOverlap": 0.8,
            "springConstant": 0.005,
        },
        "solver": "hierarchicalRepulsion",
        "minVelocity": 3,
    },
    "interaction": {
        "hover": True,
        "multiselect": True,
    },
}
net.post_initialization = """
network.on("stabilizationIterationsDone", function () {
    network.setOptions( { physics: false } );
});
"""
net.show("../Figures/ppi.html", height="100%")
```

Out[36]:

In [37]:

```
# legend for the corresponding figure in the paper
fig, ax = plt.subplots(figsize=(3, 2), dpi=200)
handles = []
for i, (domain, hexcol) in enumerate(colors.items()):
    if i % 6 == 0:
        label = "$\\bf{ADRB2}$" if i == 0 else "$\\bf{G_{α}s}$"
        item = Rectangle((0, 0), 0, 0, color="w", label=label)
        handles.append(item)
    item = Patch(color=hexcol, label=domain)
    handles.append(item)
leg = plt.legend(handles=handles, ncol=2, loc="upper left")
for vpack in leg._legend_handle_box.get_children():
    for hpack in vpack.get_children()[:1]:
        hpack.get_children()[0].set_width(0)
plt.axis('off')
fig.patch.set_facecolor('white')
fig.savefig("../Figures/ppi_legend.png", bbox_inches="tight", dpi=300)
```

In [ ]:

```

```
